# Editorial: Advances in plant-derived pharmaceuticals: exploring secondary metabolites for therapeutic innovation

**DOI:** 10.3389/fpls.2026.1976207

**Published:** 2026-09-14

**Authors:** Ramgopal Mopuri, Choon Hui Tan

**Affiliations:** 1 Department of Biochemistry, College of Agricultural Science and Applied Research, Bharatiya Engineering Science and Technology Innovation University Head Quarters, Andhra Pradesh, Sri Sathya Sai, India; 2 Department of Food Science and Nutrition, Faculty of Applied Sciences, UCSI University, Kuala Lumpur, Malaysia

**Keywords:** bioactive natural products, natural product drug discovery, plant-derived pharmaceuticals, secondary metabolites, sustainable production, therapeutic innovation

## Introduction

Plants are a rich source of structurally diverse natural compounds, and plant-derived secondary metabolites have long contributed to pharmaceutical discovery. Terpenoids, phenolics, flavonoids, alkaloids and related compounds display antioxidant, antimicrobial, anti-inflammatory and anticancer activities. The clinical history of plant-derived medicines—including quinine and vincristine—demonstrates that natural products can progress from botanical resources to important therapeutics. This Research Topic therefore addresses a timely question: how can advances in plant science, metabolite production, analytical chemistry and pharmacology make plant-derived pharmaceuticals more reproducible, sustainable and therapeutically relevant?

The path from a plant to a pharmaceutical is, however, complex. It involves plant selection and authentication, cultivation, harvesting, extraction, fractionation, purification and structural characterization, followed by quality control, pharmacological evaluation, toxicology, clinical investigation and regulatory development. Extraction may employ conventional solvents as well as Soxhlet, ultrasound-assisted, supercritical-fluid or microwave-assisted approaches, while chromatographic and spectroscopic methods are central to metabolite identification. Low abundance, matrix interference and difficulties in isolation and characterization can complicate this pipeline, while climate, soil, water availability, seasonality and postharvest handling can alter metabolite quantity and quality. These challenges make the integration of plant production with pharmaceutical quality assurance increasingly important.

This Research Topic brings together five contributions that collectively span this continuum—from environmental control of phytochemical production and horticultural optimization to phytochemical profiling, extraction technology, molecular mechanisms and therapeutic prospects. Together, they illustrate how plant-derived pharmaceutical research is moving from broad screening toward more controlled and mechanism-informed development.

## Advances in production and optimization of bioactive metabolites

The study by Debbagh-Nour et al. demonstrates that environmental conditions can differentially reshape secondary metabolism in Lavandula coronopifolia Poir. Water deficit increased total phenolic and flavonoid accumulation, whereas salinity generally reduced these compounds while increasing antioxidant activity. The findings reinforce the concept that stress should not be viewed only as a constraint on medicinal-plant production; when carefully controlled, it may also become a tool for metabolic optimization. At the same time, the reduction in biomass under severe stress highlights the need to balance metabolite concentration against total biomass yield. Such knowledge can support cultivation protocols designed to produce consistent, quality-defined medicinal raw material.

A complementary horticultural perspective is provided by Renna, who examined Taraxacum officinale L. in the context of leukemia and lymphoma research and considered how controlled cultivation, elicitation, nutrient management and spectral manipulation may enhance compounds of pharmacological interest. This cultivation-to-clinic perspective is particularly valuable because standardized pharmaceutical development ultimately depends on reproducible starting material. Future work should therefore connect cultivation variables with metabolomic fingerprints and biological potency rather than optimizing these parameters independently.

## Elucidating molecular mechanisms and therapeutic potential

Chemical identification alone does not establish therapeutic value; biological activity must be linked to plausible molecular mechanisms. Ma et al. synthesize evidence that diverse plant secondary metabolites, including flavonoids, anthraquinones, coumarins, saponins and alkaloids, can modulate autophagy-associated signaling. The review highlights pathways involving PI3K/AKT/mTOR, AMPK, Nrf2, NF-κB and related regulatory nodes. Importantly, autophagy is context-dependent: enhancing or suppressing autophagic flux may have different consequences in cancer, inflammation, neurodegeneration and metabolic disease. This emphasizes the need for dynamic autophagy measurements, target validation and dose-relevant pharmacology rather than reliance on isolated molecular markers.

The work of Renna further illustrates the value of mechanism-oriented evaluation. Preclinical evidence summarized for T. officinale indicates that metabolites such as taraxasterol, taraxacin and chicoric acid may contribute to selective cytotoxicity in leukemia and lymphoma models through oxidative stress, mitochondrial apoptosis and modulation of cancer-associated signaling. These observations are promising but remain preclinical; they should be followed by rigorous pharmacokinetic, toxicological, formulation and clinical studies before therapeutic claims can be established.

## Phytochemical profiling, extraction technology and antimicrobial potential

The contribution by Yaghoobi et al. demonstrates how phytochemical profiling can be integrated with antimicrobial, antibiofilm, antifungal and anthelmintic testing using Eryngium billardieri. The study identified diverse chemical constituents and reported biological activity against several tested microorganisms and a model nematode. Such work is relevant to the growing need for new antimicrobial leads, particularly as resistance reduces the effectiveness of existing agents. Nevertheless, extract-level activity should be interpreted cautiously: identification of active constituents, selectivity, mechanism, toxicity, pharmacokinetics and *in vivo* efficacy are necessary steps before translation.

The study by Fang et al. adds an important process-development dimension. Using Artemisia umbrosa, the authors optimized ultrasound-assisted hydrodistillation and integrated extraction efficiency with chemical characterization, odor modulation, antioxidant and antimicrobial assessment, and stability evaluation of a chamazulene-rich blue essential oil. This approach illustrates how extraction science can be linked to product quality and scalability. The broader lesson is that future plant-derived pharmaceutical research should evaluate extraction yield, chemical integrity, biological activity and stability as interconnected quality attributes.

## Nanotechnology, delivery and antimicrobial resistance

Although the five contributions do not directly evaluate nanocarrier-based delivery systems, formulation and delivery remain important future directions for poorly soluble or rapidly metabolized plant metabolites. Encapsulation, targeted delivery and controlled release could potentially improve stability and exposure, but such approaches must be evaluated alongside pharmacokinetics, toxicity, manufacturing reproducibility and regulatory requirements. The antimicrobial findings reported by Yaghoobi et al. and the antimicrobial activity examined by Fang et al. also highlight an important opportunity for plant-derived compounds in antimicrobial discovery. Future studies should prioritize pathogen-specific mechanisms, resistance development, combination strategies and clinically achievable concentrations rather than relying solely on *in vitro* inhibition.

## Future perspectives

The contributions to this Research Topic point toward a more integrated model of plant-derived pharmaceutical development. First, medicinal plants should be treated as controlled biological production systems, with genotype, environment, nutrition, irrigation, developmental stage and harvest conditions documented as critical quality variables. Second, metabolomics and advanced chromatographic and spectroscopic tools should be combined with bioassay-guided fractionation to connect chemical fingerprints with biological potency. Third, mechanistic pharmacology should be complemented by pharmacokinetics, toxicology, drug–herb interaction studies and clinically relevant formulation strategies. Fourth, sustainable cultivation and green extraction should be prioritized so that increased pharmaceutical demand does not intensify pressure on wild plant resources.

A particularly important future objective is the development of a cultivation-to-clinic framework: plant genotype and growing environment should be linked to metabolite profile, extraction process, molecular mechanism, standardized product and clinical outcome. Controlled stress and elicitation may enhance selected metabolites, but optimization must consider both metabolite yield and biomass productivity. Similarly, high biological activity *in vitro* should not be equated with clinical efficacy without evidence that the active compound or metabolite reaches the relevant tissue at an effective and safe concentration.

## Concluding remarks

The five contributions in this Research Topic collectively demonstrate that plant secondary metabolites remain a powerful platform for therapeutic innovation. They show that progress can be achieved by integrating environmental and horticultural management, phytochemical discovery, extraction engineering, molecular pharmacology and antimicrobial research. At the same time, they underscore the major translational challenges of reproducibility, standardization, bioavailability, safety and clinical validation. The future of plant-derived pharmaceuticals will depend not simply on discovering more bioactive compounds, but on developing reliable systems that connect plant biology with pharmaceutical science. Interdisciplinary collaboration among plant scientists, chemists, biotechnologists, pharmacologists, clinicians and industry will be essential to transform the chemical diversity of plants into sustainable, standardized and clinically meaningful therapeutic opportunities.

